# Structural and Functional Analysis of Validoxylamine A 7′-phosphate Synthase ValL Involved in Validamycin A Biosynthesis

**DOI:** 10.1371/journal.pone.0032033

**Published:** 2012-02-27

**Authors:** Lina Zheng, Xiang Zhou, Huaidong Zhang, Xiaofeng Ji, Lei Li, Lin Huang, Linquan Bai, Houjin Zhang

**Affiliations:** 1 Department of Biotechnology, College of Life Science and Technology, Huazhong University of Science and Technology, Wuhan, Hubei, China; 2 State Key Laboratory of Microbial Metabolism and School of Life Sciences & Biotechnology, Shanghai Jiao Tong University, Shanghai, China; 3 Yellow Sea Fisheries Research Institute, Chinese Academy of Fishery Sciences, Qingdao, Shandong, China; Russian Academy of Sciences, Institute for Biological Instrumentation, Russian Federation

## Abstract

Validamycin A (Val-A) is an effective antifungal agent widely used in Asian countries as crop protectant. Validoxylamine A, the core structure and intermediate of Val-A, consists of two C_7_-cyclitol units connected by a rare C-N bond. In the Val-A biosynthetic gene cluster in *Streptomyces hygroscopicus* 5008, the ORF *valL* was initially annotated as a validoxylamine A 7′-phosphate(V7P) synthase, whose encoded 497-aa protein shows high similarity with trehalose 6-phosphate(T6P) synthase. Gene inactivation of *valL* abolished both validoxylamine A and validamycin A productivity, and complementation with a cloned *valL* recovered 10% production of the wild-type in the mutant, indicating the involvement of ValL in validoxylamine A biosynthesis. Also we determined the structures of ValL and ValL/trehalose complex. The structural data indicates that ValL adopts the typical fold of GT-B protein family, featuring two Rossmann-fold domains and an active site at domain junction. The residues in the active site are arranged in a manner homologous to that of *Escherichia coli (E.coli)* T6P synthase OtsA. However, a significant discrepancy is found in the active-site loop region. Also noticeable structural variance is found around the active site entrance in the apo ValL structure while the region takes an ordered configuration upon binding of product analog trehalose. Furthermore, the modeling of V7P in the active site of ValL suggests that ValL might have a similar SNi-like mechanism as OtsA.

## Introduction

Aminocyclitols are a diverse class of bioactive compounds produced by the *Streptomyces* genus [1]. Based on the chemical structures, aminocylitols can be divided into several groups, such as aminoglycoside, C_7_N-aminocyclitols and five-membered ring aminocyclitols [2]. The C_7_N-aminocyclitols, including acarbose, pyralomicin, salbostatin, cetoniacytone A and Val-A, have gained increasing attention due to their extensive applications in agriculture and medicine. Val-A is an antifungal agent isolated from *S. hygroscopicus var. limoneus* and *S. hygroscopicus var. jinggangensis* 5008. It is used in many Asian countries as a crop protectant against *Rhizoctonia solani*, which is a pathogenic fungus responsible for various diseases in plants [3]. The antifungal capability of Val-A is attributed to its ability to inhibit trehalase, a trehalose-hydrolyzing enzyme. The inhibition of trehalase disrupts the carbon supply and energy production in the fungi, which leads to growth retardation and death of the pathogen [4].

**Figure 1:**
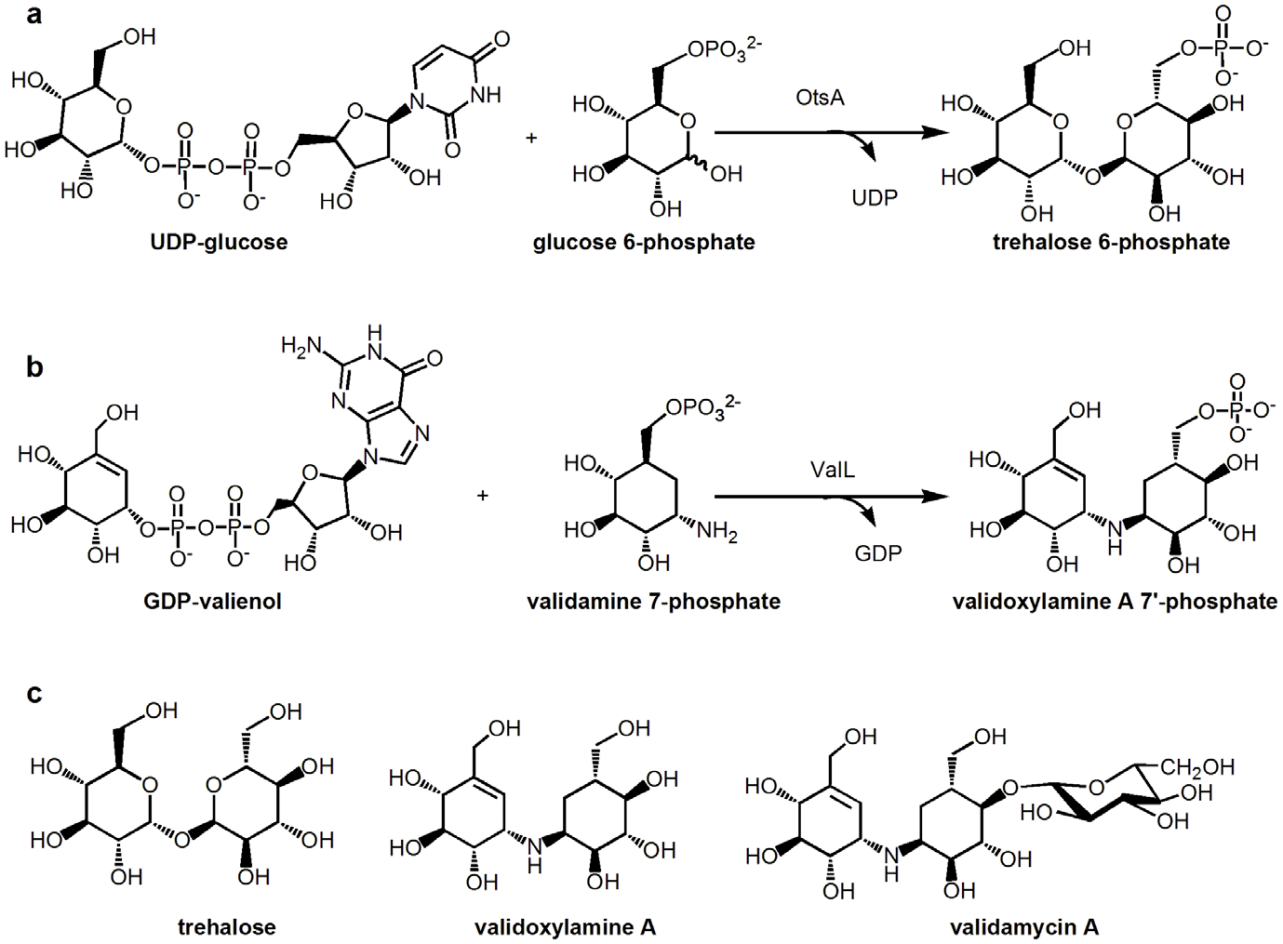
Reactions catalyzed by OtsA, ValL and chemical structures of related natural products. (a) The reaction catalyzed by OtsA. (b) The reaction catalyzed by ValL. (c) Chemical structures of validoxyamine A, trehalose and validamycin A.

The structure of Val-A consists of an unsaturated valienol unit connected to a saturated validamine unit through a 1-1 α bond, and a glucose unit attached to the validamine moiety [5]. Feeding experiments indicated the 7-carbon unit in the Val-A structure originates from the cyclization of sedoheptulose 7-phosphate [1]. Validamycin biosynthetic gene clusters had been independently cloned from two above-mentioned producers [6], [7]. The heterologous expression of eight genes, *valABCGKLMN* with *valL* annotated as V7P synthase gene, rendered the surrogate host with validamycin productivity [7]. Through multiple steps, the 2-*epi*-5-*epi*-valiolone is proposed to be converted to NDP-valienol and validamine 7-phosphate [7], [8], [9], [10], [11]. Subsequently, the rare nitrogen-bridged twin-cyclitol core structure in Val-A arises from the condensation of NDP-valienol and validamine 7-phosphate (Figure 1). This coupling reaction was proved to be catalyzed by the pseudoglycosyltransferase ValL, also named as VldE in the biosynthetic gene cluster from *S. hygroscopicus var. limoneus* [7], [12]. As a result, a rare nonglycosidic C-N bond is formed between valienol and validamine units with the retention of anomeric configuration in each cyclitol ring. Several other natural products in the aminocyclitol group also contain the unusual C-N bond, such as acarbose, adiposin-1, salbostatin, trestatin, amylostatins and pyralomicin [1], [13], [14]. In addition to ValL/VldE, AcbS/GacS, from *Actinoplanes sp*. *SE50/110* and *Streptomyces glaucescens* respectively, are also thought to be responsible for the C-N bond formation in acarbose biosynthesis [15].

**Figure 2:**
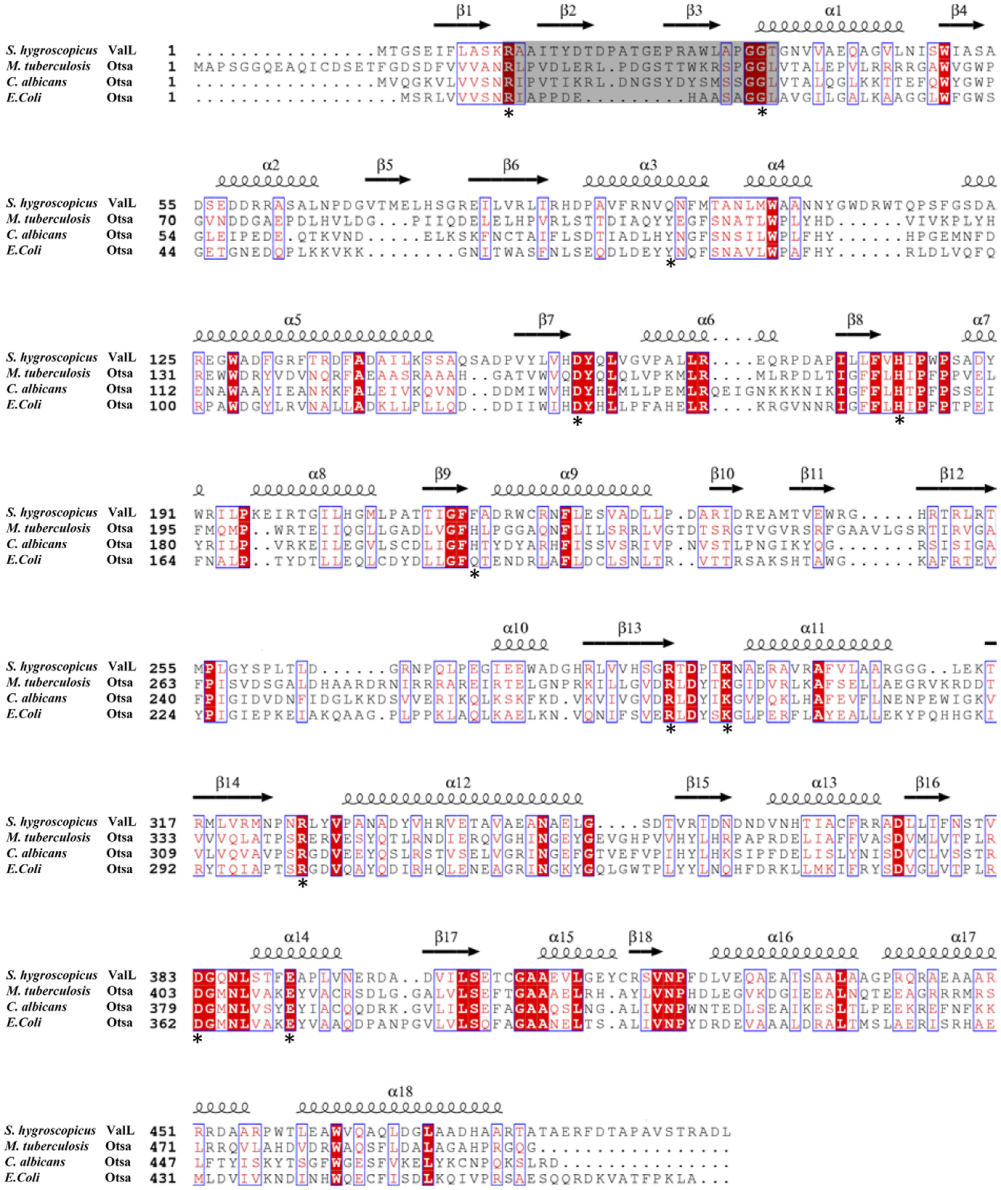
Structure-based sequence alignment of ValL and OtsA from several species. The primary sequence of ValL is aligned with the OtsA sequences from *E. coli*, *Mycobacterium tuberculosis*, and *Candida albicans*. The sequences are annotated with corresponding secondary structures in ValL. Arrows represent β-sheets and helices represent α-helices. The active-site loops are shaded and the conserved residues are colored in red. The residues involved in binding of V7P to OtsA are marked by 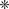.

Due to the sequence similarity between ValL and *E.coli* T6P synthase OtsA, ValL was regarded as T6P synthase with relaxed specificity (Figure 2). However, recent biochemical experiments have shown that ValL/VldE has no T6P synthase activity. It has strict substrate specificity for GDP-valienol and validamine 7-phosphate. The replacement of either cyclitol derivative with a glucose analog abolishes the reaction [12].

Although the catalytic activity of ValL/VldE has been studied, genetic evidence for its involvement in Val-A biosynthesis is not available, and the structural aspect of the protein activity remains unexplored. In this report, we present *in vivo* gene inactivation and complementation of *valL*, and the crystal structures of apo ValL and ValL/trehalose complex. The inactivation and complementation of *valL* indicates *valL* is an essential part of the Val-A biosynthesis pathway. The 1.7 Å crystal structure shows that the binding site for V7P is well conserved in ValL and OtsA while the binding site for the nucleotide substrate is different. The modeling of V7P in the active site of ValL suggests that ValL might have a similar SNi-like mechanism as OtsA.

## Results and Discussion

### Inactivation of *valL* abolishes validamycin A production

**Figure 3:**
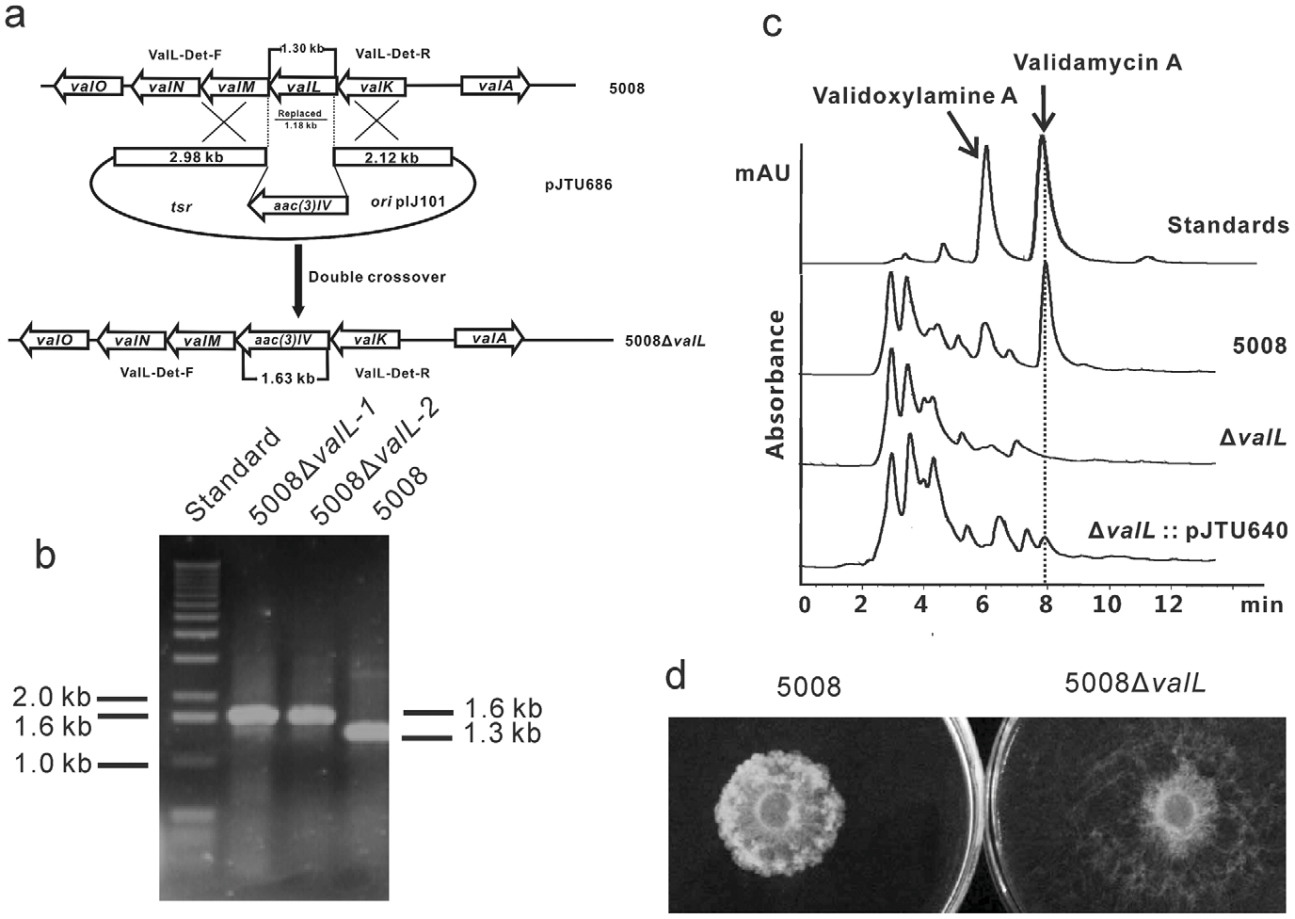
Inactivation of *valL* and complementation of the mutant. (a) Schematic representation of the replacement of an 1.18-kb internal fragment of *valL* with the 1.4-kb *aac(3)IV*. In shuttle plasmid pJTU685, *aac(3)IV* was inserted between the 2.98-kb and 2.12-kb genomic fragments originally flanking the deleted 1.18-kb region. While wild-type *S. hygroscopicus* 5008 should give a 1.30-kb PCR-amplified product, mutant 5008Δ*valL* should yield a 1.60-kb product by using a pair of primers, valL-Det-F and valL-Det-F. (b) PCR analysis of wild-type *S. hygroscopicus* 5008 and *valL* mutant. (c) HPLC profiles of the standards, 5008, 5008Δ*valL*, and 5008Δ*valL*::pJTU640. Plasmid pJTU640 is an integrative plasmid cloned with intact *valL* under the control of PermE* promoter. (d) Bioassay comparison between the 5008 and 5008Δ*valL*. One mL of fermentation supernatant was mixed with 14 mL of melted 0.8% agar. An agar plug with the fungal indicator *Pellicularia sasakii* was transferred to the center of the agar plate. After 24 h incubation at 30°C, the diameter of the colony was measured, which is inversely related to the inhibitory potency.

In order to genetically prove the involvement of *valL* in Val-A biosynthesis, a 1.18-kb internal region of *valL* was replaced by an *aac(3)IV* cassette in strain 5008. This was achieved by using a pHZ1358-derived plasmid pJTU685, in which *valL* had been replaced by *aac(3)IV* between a 2.98-kb left flanking and a 2.12-kb right flanking sequences of *valL* (Figure 3a). The plasmid pJTU685 was introduced into strain 5008 through conjugation, and thiostrepton-sensitive and apramycin-resistant exconjugants were selected. The mutants 5008Δ*valL* were further confirmed by PCR amplification. The *valL* mutant gave an expected 1.60-kb PCR product, whereas the wild-type strain 5008 gave a 1.30-kb product (Figure 3b). Fermentation broths of the mutants were analyzed by HPLC and bioassay. No peak corresponding to Val-A and validoxylamine A was detected by HPLC analysis (Figure 3c), and inhibition of the fungus *Pellicularia sasakii* could not be observed in the bioassay (Figure 3d), indicating a complete loss of production of both compounds in the *valL* mutants.

When a pPM927-derived integrative plasmid pJTU640, with an intact *valL* under the control of PermE* promoter, was introduced into 5008Δ*valL*, the culture broth of the thiostrepton-resistant exconjugant was found to regain the productivity of Val-A and validoxylamine A by HPLC analysis (Figure 3c). Both inactivation and complementation experiments clearly demonstrated that valL is essential for the biosynthesis of Val-A and its intermediate validoxylamine A.

### Overall structure of ValL

**Figure 4:**
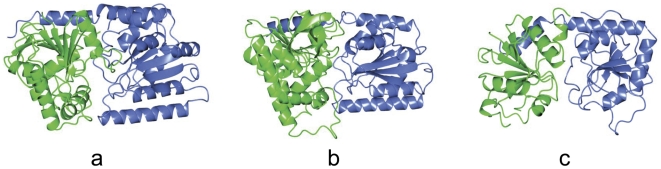
Three-dimensional structures of ValL and GT-B glycosyltransferases. A diagram showing N-terminal domain (green), C-terminal domain (blue) and the cleft harboring the active site. (a) ValL. (b) OtsA. (c) T4 β-glucosyltransferase.

The overall structure of ValL closely resembles the classic fold of “GT-B” glycosyltransferases (Figure 4). It harbors two domains, each of which contains a β/α/β Rossmann fold, and an active site at their interface. The N-terminal domain of ValL (1–263) consists of twelve β-strands flanked by nine helices. The C-terminal domain (264–481), which adopts a similar fold, consists of six parallel β-strands surrounded by eight helices. Also found in the ValL structure is a kink between last two C-terminal helices, which is the common feature of GT-B enzyme family. The kink directs the last C-terminal helix into the vicinity of the N-terminal domain and causes the residues in the helix to interact with the N-terminal domain. ValL exists as a dimer in the crystal and the dimerization interface, which lies at the opposite side of the active site, covers a wide region on the surface (the buried area 10,834 Å^2^). The dynamic scattering experiment indicated that ValL forms a dimer in solution(data not shown). It is unclear why ValL forms a dimer. In other organisms, such as *Saccharomyces cerevisae*, the T6P synthase and phosphatase form a multiunit complex [16]. It is possible that the dimerization of the enzyme could facilitate the formation of the ValL/phosphatase complex which might be important for its function.

### Comparison between OtsA and ValL Structures

**Figure 5:**
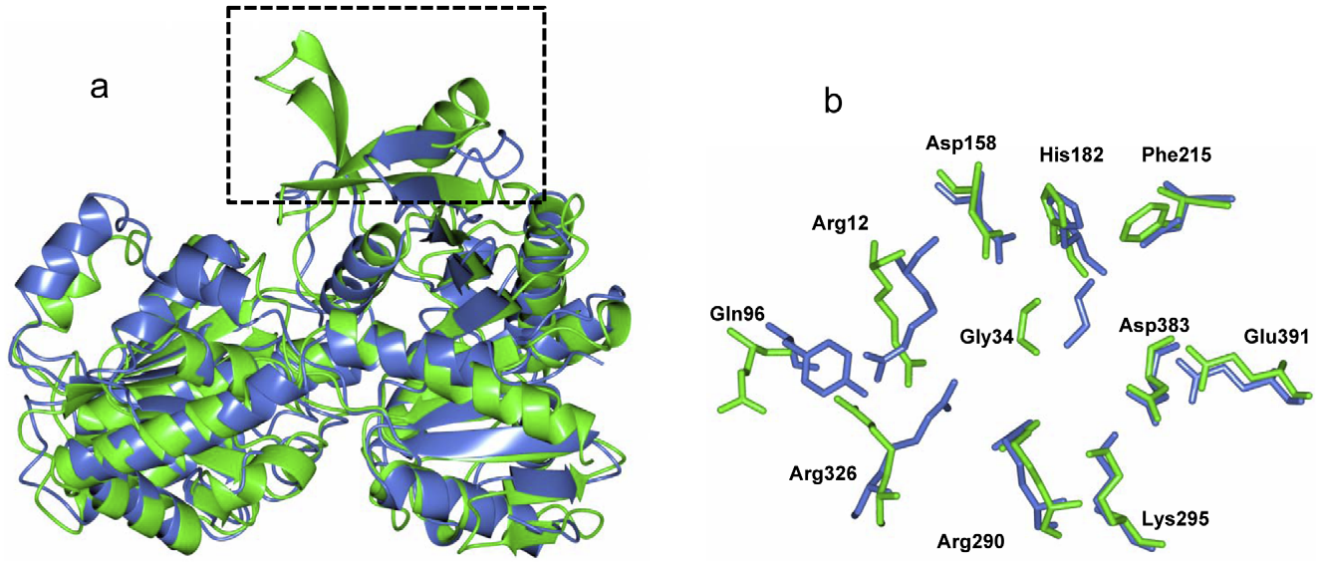
Superposition of OtsA and ValL structures. (a) Ribbon diagram overlap. The ribbon diagram of OtsA (colored in blue) is superposed with that of ValL(colored in green). The boxed region shows the main difference between two structures. (b) Active site overlap. The active site residues responsible for V7P binding in OtsA (colored in blue) are superposed with the active site residues in ValL(colored in green). Residue-labels in ValL are shown.

The overall structure of ValL is analogous to that of OtsA. The secondary structure matching between these two structures resulted in a root mean square deviation(RMSD) of 1.83 Å over 369 residues. A notable difference lies in the loop region ranging from Arg12 to Thr35 (Figure 2). As the shortest loop in the GT-B protein family, the active site loop in OtsA structure is located within the cleft between N-terminal and C-terminal domains. In the ValL structure, due to a 9-residue insertion, the active-site loop protrudes out of the cleft and forms part of a 12-strand β-sheet at N-terminal domain. Furthermore, the β5 and β6 strands of ValL are significantly larger than the counterparts in OtsA. The α2 helix is unique for ValL as the corresponding sequence in OtsA forms a flexible loop(Figure 5a). The residues responsible for the binding of V7P in OtsA are mostly conserved in ValL(Figure 5b). In contrast, the residues within hydrogen bonding distance to the uridine moiety are different, which is consistent with ValL's preference for GDP-valienol as its substrate. Although the nucleosides in ValL and OtsA substrates are distinctive, the replacement of UDP-glucose with GDP-glucose does not render ValL with T6P synthase activity [12]. Therefore, nucleoside is not the determinant for ValL and OtsA substrate specificity.

### Binding of trehalose and modeling of validoxylamine A 7′-phosphate in the active site

**Figure 6:**
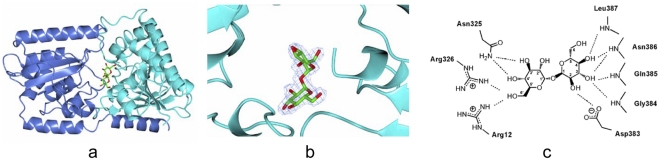
Close-up view of the active site with a trehalose molecule in it. (a) A ribbon diagram showing a trehalose molecule binds in the active site located at the junction of N-terminal (cyan) and C-terminal (blue) domains. (b) Close-up view of the trehalose binding site. The electron density map(in blue) is part of an omit map calculated with coefficients |Fobs| -|Fcalc| and phases from an omit model of ValL/trehalose complex without the trehalose molecule. The map has 1.7 Å resolution and is contoured at 3σ level. (c) Hydrogen bond network formed between the trehalose molecule and the residues around it.

To probe the mechanism of reaction catalyzed by ValL, its product analogs, trehalose and validoxylamine A, were used to form complex with ValL. The co-crystallization of ValL/trehalose led to the determination of ValL/trehalose complex structure. The electron density at the junction of N-terminal and C-terminal domains unambiguously indicates the location of trehalose embedded in the active site (Figure 6a & 6b). The location of trehalose allows clear description of interaction between the trehalose molecule and ValL. The trehalose molecule associates with the active site through a network of hydrogen bonds (Figure 6c). The hydroxyl group on trehalose C6′ interacts with guanidine side chains of Arg12, Arg326; the hydroxyl groups on C3′ and C4′ form hydrogen bonds with Asn325 amide side chain; the hydroxyl groups on C2 form hydrogen bond with Asp383 side chain carboxylate. Also the binding of trehalose molecule is maintained through the interaction between the exocyclic hydroxyl groups and the protein backbone. The hydroxyl group at C3 position forms hydrogen bonds with the backbone amide groups on Gly384, Gln385, Asn386; the hydroxyl group at C4 position forms hydrogen bonds with backbone amide groups on Asn386 and Leu387.

**Figure 7:**
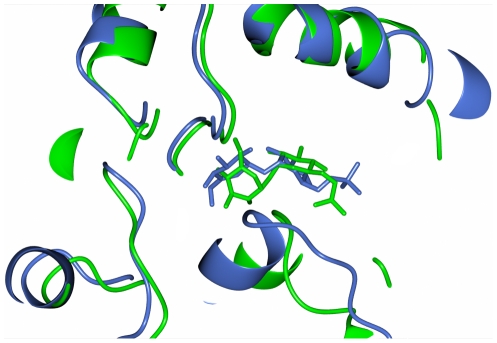
Modeling of V7P in the ValL active site. The V7P molecule is modeled in the active site of ValL using the docking program AutoDock. The ValL/V7P complex model (colored in green) is superposed with OtsA/V7P complex structure (colored in blue). The V7P molecules are shown in cylinders and the proteins are shown in ribbons.

**Figure 8:**
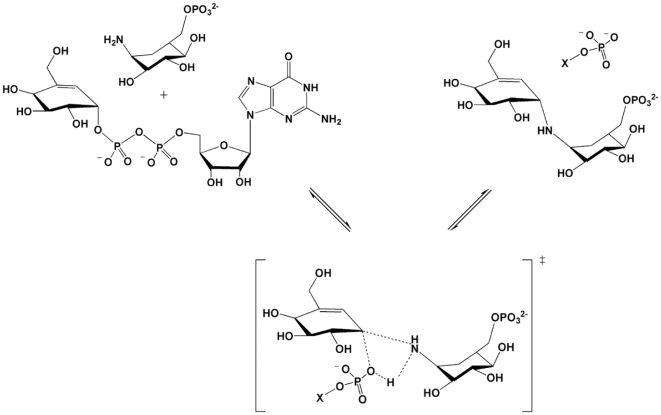
The proposed mechanism for ValL. Adopted from the mechanism of OtsA, a SNi-like mechanism is proposed for ValL. The transition state is enclosed in brackets and GMP is designated as X.

As multiple attempts to crystallize ValL/validoxylamine A complex proved to be fruitless, the V7P molecule was modeled into the active site of ValL to illustrate the binding site of its natural product. The modeling result indicates apo ValL active site could accommodate V7P molecule readily. The superposition of ValL/V7P complex and OtsA/V7P complex structures shows that V7P adopts similar positions in both active sites (Figure 7). A SNi-like mechanism was proposed for the reaction catalyzed by ValL [12]. The oxocarbenium-like transition state involves the carbonyl oxygen on the protein backbone and GDP-valienol. Based on OtsA/V7P structure, a slightly different SNi-like mechanism was proposed for V7P synthesis [17], [18]. As illustrated by the hydrogen bond formed between donor phosphate oxygen O3B and bridging NH on V7P, a transition state is formed with C1 carbon on cyclohexenyl moiety, NH_2_ group on validamine 7-phosphate and O3B on donor phosphate [17]. As the active site residues are highly conserved between OtsA and ValL, the mechanism of ValL is likely to be homologous to that illustrated by the OtsA/V7P complex structure(Figure 8).

### Structural change induced by the binding of trehalose

**Figure 9:**
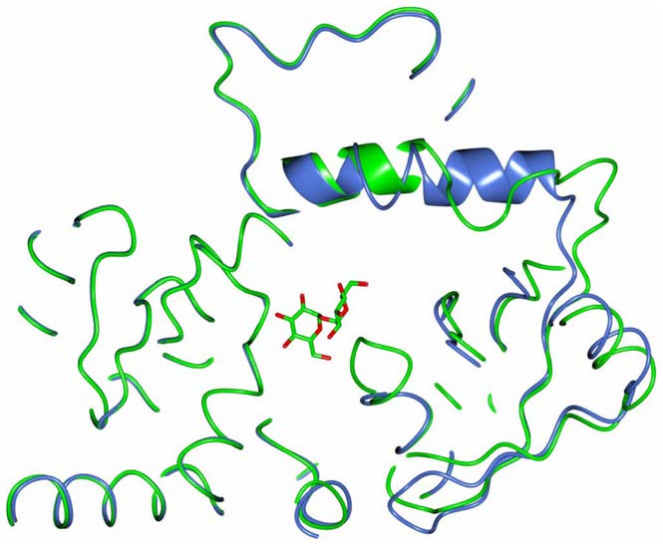
Structural variance in the region encompassing 88–110 residues of ValL. ValL/trehalose complex structure and ValL apo structure are superposed with each other. The 88–110 regions in chain B of ValL/trehalose complex structure(in blue) and chain B of ValL apo structure (in green) are shown as ribbons. The rest of the molecules are shown as worms.

The structure of apo ValL contains two molecules in the asymmetric unit. The SSM alignment shows the backbone of chain A and chain B matches well with an RMSD of 0.606 Å across 461 Cα atoms. However, a significant discrepancy has been observed in the region encompassing residue 88–110 (Figure 9). In chain A, these residues form two adjacent helices. In contrast, in chain B, this region consists of an N-terminal loop and a C-terminal α-helix. Interestingly, in the ValL/trehalose complex structure, the folding of residues 88–110 is exactly the same as that in chain A of apo ValL structure. This region forms part of the acceptor binding site. It is possible that this part of the protein folds in different ways and once the active site is occupied by the ligand, the ligand forces the surrounding residues to form an ordered folding as in the ValL/trehalose complex structure. The ligand-induced structural change has been observed in other GT-B glycosyltransferases. In OtsA, significant change can be observed in the active site loop and adjacent region when the associated ligand was changed from glucose-6-phosphate to UDP-glucose. The structural change observed in OtsA and ValL is restricted in the acceptor domain while the relative orientation of the donor and acceptor domains stays the same [19]. In contrast, the binding of UDP in phage T4 β-glucosyltransferase induces a 14-degree domain rotation which results in a closed conformation [20]. It is noticeable that all the OtsA structures available are protein/ligand complex structures [17], [19], [21]. The apo structure of ValL thus demonstrates for the first time the structural characteristics of this class of retaining enzymes in the apo form, which sheds light on the conformational changes accompanying substrate binding and enzyme activation.

**Figure 10:**
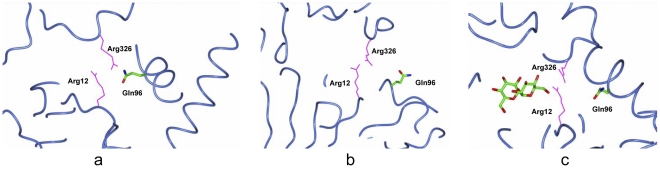
Change of orientation at Gln96 upon occupation of the active site. (a) In the structure of apo ValL, the region encompassing residues 88–110 on chain A has a closed configuration. As a result, the residue Gln96 adopts an orientation pointing towards the active site which is marked by Arg12 and Arg326. (b) On chain B of apo ValL, the region 88–110 takes an open configuration and the residue Gln96 points away from the active site (c) In the structure of ValL/trehalose complex, the segment of residues 88–110 takes a single closed configuration with Gln96 pointing towards the active site.

Although V7P binding residues in OtsA are conserved in ValL, Tyr 76 in OtsA is an exception. In the OtsA/V7P complex structure, Tyr76 is responsible for binding of the acceptor phosphate group and positioning the Arg9 to accommodate the substrate [21]. Based on the sequence alignment, Gln96 in ValL corresponds to Tyr76 in OtsA (Figure 2). Interestingly, Gln96 in ValL adopts two positions in the apo form, one pointing towards active site (Figure 10a) and one pointing away from active site(Figure 10b). However, in the presence of trehalose, Gln96 takes an ordered position toward the trehalose molecule(Figure 10c). As a result, it forms a water-bridged hydrogen bond with Arg326 which is responsible for the substrate binding. It is noteworthy that Gln96 lies at the entrance of the active site and the surrounding region(residues 88–110) takes alternative configuration upon trehalose binding. It is possible that this region serves as a gate which is responsible for opening and closing of the active site.

## Materials and Methods

### Bacterial strains, media, and cultivation

*S. hygroscopicus var. jinggangensis* 5008 and its derivatives were grown either on solid SFM medium or in modified liquid TSBY medium (with 10.3% sucrose). Fermentation was carried out at 37°C for 5 days. *E. coli* strains were cultivated at 37°C in Luria-Bertani medium or on Luria-Bertani agar plates. *E. coli* DH10B (GIBCO-BRL), ET12567 (pUZ8002) [22] and BW25113 (pIJ790) [23] were used as *E. coli* hosts for plasmid construction, *E. coli*-*Streptomyces* conjugation, and ReDirect technology, respectively [23].

### Construction and complementation of the *valL*-disrupted mutant

The inactivation of gene *valL* was achieved by replacing with a cassette of apramycin-resistant marker *aac(3)IV* using a PCR-based ReDirect technology [23]. A 6.3-kb BclI fragment containing *valL* was cloned to pHZ1358 [24] and generated pJTU685. The cassette was amplified using plasmid pIJ773 as template and a pair of primers valL-targeting-F (5′-GCCAACCTGATGTGGGCGGCGAACAACTACGGCTGGGACattccggggatccgtcgacc-3′) and valL-targeting-R (5′-TCAGAGGTCTGCTCGTGTCGAGACTGCCGGTGCGGTGTCtgtaggctggagctgcttc-3′), and pIJ773 homologous sequences were in lowercase. *E. coli* BW25113/pIJ790 bearing pJTU685 was electroporated with the amplified cassette. Mutated plasmid was firstly introduced into non-methylating *E. coli* ET12567 pUZ8002) through transformation and then transferred into *S. hygroscopicus* 5008 by intergeneric conjugation [9]. Exconjugants with double cross-over were selected for their apramycin resistance and named as LL2. The allelic replacement of *valL* in the LL2 mutant was confirmed by PCR amplification with the primers of valL-Det-F (5′-CCGGCGTCCTCAACATC-3′) and valL-Det-R (5′ -TGCTCGTGTCGAGACTGC-3′).

For the complementation of the mutant, PCR-amplified *valL* was cloned into pJTU968 [11] through EcoRI and NdeI digestions to generate pJTU634. A 1.7-kb fragment with *valL* was cleaved by EcoRI and MunI and cloned into pPM927 digested with EcoRI [25]. The new plasmid, pJTU640, was introduced into LL2 through conjugation, and exconjugants were selected with 25 µg/mL thiostrepton. The exconjugants were named as LL2-1 and investigated through fermentation, HPLC analysis, and bioassay as previously described [9].

### Expression and purification of ValL

Gene *valL* was cloned into pET28a vector between EcoRI and BamHI recognition sites. The pET28a-ValL plasmid was transformed into *E. coli* BL21(DE3), and a single colony was used to inoculate a 10 mL overnight culture containing 50 µg/mL kanamycin. Then the culture was used to inoculate 3L LB broth, and the culture was grown at 37°C until OD_600_ reached 0.5. The protein expression was induced with 0.3 mM Isopropyl β-D-1-thiogalactopyranoside (IPTG), and the incubation was continued for another 12 hours at 18°C. Cells were collected by centrifugation at 6,000 g, 4°C for 10 minutes. The resulting cell pellet was resuspended in 30 mL buffer containing 20 mM Tris, 0.5 M NaCl, pH 7.9. The cells were lysed with French Press, and the cellular debris was removed by centrifugation at 40,000 g for 20 minutes. The recombinant ValL was purified by chromatography with Ni-NTA resin. The bound protein was washed with wash buffer (30 mM imidazole, 20 mM Tris, 0.5 M NaCl, pH 7.9) and eluted with elution buffer (200 mM Imidazole, 20 mM Tris, 0.5 M NaCl, pH 7.9). The protein content in each fraction was resolved on sodium dodecyl sulfate (SDS) gel before appropriate fractions were pooled and further purified with size-exclusion chromatography on a Superdex 75 column (16/60,G. E. Healthcare) equilibrated with 0.5 M NaCl, 20 mM Tris, PH 7.5. The protein was then concentrated with Amicon® Ultra-4 Centrifugal Filter Units, and the concentration was estimated spectrometerically at OD_280_. Selenomethionine-labeled ValL was expressed as described in the literature and purified as native protein [26]. In brief, 5 ml of overnight culture was used to inoculate 3L M9 medium supplemented with 50 µg/mL kanamycin, 2 mM MgSO_4_, 0.1 mM CaCl_2_, and 5 g/L glucose. The culture was grown at 37°C until OD_600_ reached 0.6. Then lysine, phenylalanine, and threonine were added at 100 mg/L, isoleucine, leucine, and valine were added at 50 mg/L, and L-selenomethionine was added at 60 mg/L. After incubation for 15 min, 0.3 mM IPTG was added to induce the protein expression and the incubation was continued for another 12 hours at 18°C.

### Crystallization and data collection

Crystals of native ValL were grown by the hanging-drop vapor-diffusion method. Briefly, 1 µL of protein (10 mg/mL) was mixed with the precipitant solution containing 30% (w/v) polyethylene glycol 4000, 200 mM MgCl_2_, 100 mM Tris-HCl (pH8.5). The mixture was allowed to equilibrate over a well filled with 150 µL precipitant solution. The crystals appeared after overnight incubation at 20°C and reached their maximum size three days later. Selenomethionine-labeled ValL crystals were grown in the same manner. To obtain ValL/trehalose complex structure, 5 mM trehalose was incubated with the enzyme for 30 min prior to mixing with the precipitant solution. A cryo-protectant solution was made by supplementing precipitant solution with 30% trehalose. The crystals were immersed in the cryoprotectant briefly before frozen in liquid nitrogen.

Diffraction data were collected at 100 k from selenomethionine-labeled ValL crystals at the selenium absorption edge with a Mar 350 CCD detector on beam-line BL17U, Shanghai Synchrotron Radiation Facility (SSRF), Shanghai, China. The data set was indexed with XDS package and scaled with Scala [27], [28]. Initial phases were obtained by single-wavelength anomalous diffraction (SAD) using the anomalous scattering caused by the selenomethionine incorporated at the methionine sites. All 16 selenium sites in the asymmetric unit were located with PHENIX package and the phase was refined with a figure of merit of 0.42 to 2 Å [29]. The phase was further improved with DM program in the CCP4 package(figure of merit = 0.74) [30]. The resulting electron density map was of very good quality and side-chains of 80% residues could be automatically built with PHENIX [29]. The manual building was done with Coot and the structure was refined with PHENIX after each building cycle [31]. Portion of the data (5%) was set aside to calculate free R factor, which was used to monitor the bias throughout the model building process [32]. The stereochemistry of the model was validated at the late stage of manual building with MolProbity [33]. Native data set was collected at 100 k with a Mar 165 detector on beam-line 3W1A, Beijing Synchrotron Radiation Laboratory (BSRF), Beijing, China. The native structure was solved by molecular replacement with Phaser, using semet ValL structure as the search model [34]. Data parameters and refinement statistics are summarized in Table 1. The V7P molecule was modeled into apo ValL structure using program AutoDock4.2 [35]. Autodock Tools was used to preparing the protein and ligand. The grid box size was set at 70×70×70 Å. The center (−0.920, 10.832, 57.006) of the box was chosen according to the position of key residues in the pocket. All of the docking decoys were clustered with a cut-off limit of 2 Å according to RMSD.

**Table 1:** Data collection and refinement statistics.

	Native ValL	SeMet ValL
Data collection		
Space group	P2_1_	P2_1_
Cell dimensions		
a, b, c (Å)	80.7, 46.7, 123.9	79.7, 45.8, 115.0
α, β, γ(°)	90.0, 108.1, 90.0	90.0, 107.36, 90.0
Resolution (Å)^1^	40.96–1.7(1.79–1.7)	45.8–1.7(1.79–1.7)
Wavelengths(Å)	0.9792	0.9792
Rmerge	0.083(0.552)	0.058(0.718)
I/σ(I)	22.2(5.0)	17.2(2.1)
Completeness (%)	97.7(87.6)	99.1(94.2)
Redundancy	7.2(6.4)	7.1(5.9)
Wilson B-factors(Å^2^)	13.0	23.6
Anomalous completeness		98.5(92.5)
FOM/DM FOM^2^		0.42/0.74
Refinement		
Resolution (Å)	40.96–1.7(1.79–1.7)	45.8–1.7(1.79–1.7)
No. reflections	90507	82832
R_work_/R_free_ 3	0.179/0.209	0.162/0.214
Number of atoms		
Protein	7387	7181
Trehalose		46
Water	828	627
Average B-factor (Å^2^)		
Protein	9.96	30.61
Trehalose		21.62
Water	21.64	40.21
R.m.s deviations		
Bond lengths (Å)	0.009	0.011
Bond angles (°)	1.221	1.432
Ramachandran plot^4^		
Favored region (%)	98.19	97.12
Allowed region (%)	1.49	2.47
Outliers (%)	0.32	0.41
PDB code	3T5T	3T7D

1 Data in the parenthesis was calculated based on the highest resolution shell.

2 Mean figure of merit with or without density modification.

3 R-factor = (Σhkl||Fo|-|Fc||)/Σhkl|Fo| where Fo and Fc are the observed and calculated structure factors respectively. R_free_ was calculated with a randomly-selected 5% subset which was excluded from the refinement process.

4 Statistics of Ramachandran plot were calculated with MolProbity.

### Data deposition

The atomic coordinates and the structure factors of apo ValL and ValL/trehalose complex have been deposited in the Protein Data Bank (www.rcsb.org) with the accession codes of 3T5T and 3T7D respectively.

## References

[1] Mahmud T (2003). The C7N aminocyclitol family of natural products.. Nat Prod Rep.

[2] Mahmud T (2009). Progress in aminocyclitol biosynthesis.. Curr Opin Chem Biol.

[3] Iwasa T, Yamamoto H, Shibata M (1970). Studies on validamycins, new antibiotics. I. Streptomyces hygroscopicus var. limoneus nov. var., validamycin-producing organism.. J Antibiot (Tokyo).

[4] Asano N, Yamaguchi T, Kameda Y, Matsui K (1987). Effect of validamycins on glycohydrolases of Rhizoctonia solani.. J Antibiot (Tokyo).

[5] Suami T, Ogawa S, Chida N (1980). The revised structure of validamycin A.. J Antibiot (Tokyo).

[6] Singh D, Seo MJ, Kwon HJ, Rajkarnikar A, Kim KR (2006). Genetic localization and heterologous expression of validamycin biosynthetic gene cluster isolated from Streptomyces hygroscopicus var. limoneus KCCM 11405 (IFO 12704).. Gene.

[7] Bai L, Li L, Xu H, Minagawa K, Yu Y (2006). Functional analysis of the validamycin biosynthetic gene cluster and engineered production of validoxylamine A.. Chem Biol.

[8] Dong H, Mahmud T, Tornus I, Lee S, Floss HG (2001). Biosynthesis of the validamycins: identification of intermediates in the biosynthesis of validamycin A by Streptomyces hygroscopicus var. limoneus.. J Am Chem Soc.

[9] Yu Y, Bai L, Minagawa K, Jian X, Li L (2005). Gene cluster responsible for validamycin biosynthesis in Streptomyces hygroscopicus subsp. jinggangensis 5008.. Appl Environ Microbiol.

[10] Minagawa K, Zhang Y, Ito T, Bai L, Deng Z (2007). ValC, a new type of C7-Cyclitol kinase involved in the biosynthesis of the antifungal agent validamycin A.. Chembiochem.

[11] Xu H, Zhang Y, Yang J, Mahmud T, Bai L (2009). Alternative epimerization in C(7)N-aminocyclitol biosynthesis is catalyzed by ValD, a large protein of the vicinal oxygen chelate superfamily.. Chem Biol.

[12] Asamizu S, Yang J, Almabruk KH, Mahmud T (2011). Pseudoglycosyltransferase catalyzes nonglycosidic C-N coupling in validamycin a biosynthesis.. J Am Chem Soc.

[13] Yokose K, Ogawa K, Suzuki Y, Umeda I, Suhara Y (1983). New alpha-amylase inhibitor, trestatins. II. Structure determination of trestatins A, B and C.. J Antibiot (Tokyo).

[14] Kawamura N, Sawa R, Takahashi Y, Isshiki K, Sawa T (1996). Pyralomicins, novel antibiotics from Microtetraspora spiralis. II. Structure determination.. J Antibiot (Tokyo).

[15] Wehmeier UF, Piepersberg W (2004). Biotechnology and molecular biology of the alpha-glucosidase inhibitor acarbose.. Appl Microbiol Biotechnol.

[16] Bell W, Sun W, Hohmann S, Wera S, Reinders A (1998). Composition and functional analysis of the Saccharomyces cerevisiae trehalose synthase complex.. J Biol Chem.

[17] Errey JC, Lee SS, Gibson RP, Martinez Fleites C, Barry CS (2010). Mechanistic insight into enzymatic glycosyl transfer with retention of configuration through analysis of glycomimetic inhibitors.. Angew Chem Int Ed Engl.

[18] Lee SS, Hong SY, Errey JC, Izumi A, Davies GJ (2011). Mechanistic evidence for a front-side, SNi-type reaction in a retaining glycosyltransferase.. Nat Chem Biol.

[19] Gibson RP, Tarling CA, Roberts S, Withers SG, Davies GJ (2004). The donor subsite of trehalose-6-phosphate synthase: binary complexes with UDP-glucose and UDP-2-deoxy-2-fluoro-glucose at 2 A resolution.. J Biol Chem.

[20] Morera S, Lariviere L, Kurzeck J, Aschke-Sonnenborn U, Freemont PS (2001). High resolution crystal structures of T4 phage beta-glucosyltransferase: induced fit and effect of substrate and metal binding.. J Mol Biol.

[21] Gibson RP, Turkenburg JP, Charnock SJ, Lloyd R, Davies GJ (2002). Insights into trehalose synthesis provided by the structure of the retaining glucosyltransferase OtsA.. Chem Biol.

[22] Paget MS, Chamberlin L, Atrih A, Foster SJ, Buttner MJ (1999). Evidence that the extracytoplasmic function sigma factor sigmaE is required for normal cell wall structure in Streptomyces coelicolor A3(2).. J Bacteriol.

[23] Gust B, Challis GL, Fowler K, Kieser T, Chater KF (2003). PCR-targeted Streptomyces gene replacement identifies a protein domain needed for biosynthesis of the sesquiterpene soil odor geosmin.. Proc Natl Acad Sci U S A.

[24] Sun Y, He X, Liang J, Zhou X, Deng Z (2009). Analysis of functions in plasmid pHZ1358 influencing its genetic and structural stability in Streptomyces lividans 1326.. Appl Microbiol Biotechnol.

[25] Smokvina T, Mazodier P, Boccard F, Thompson CJ, Guerineau M (1990). Construction of a series of pSAM2-based integrative vectors for use in actinomycetes.. Gene.

[26] Doublie S (2007). Production of selenomethionyl proteins in prokaryotic and eukaryotic expression systems.. Methods Mol Biol.

[27] Kabsch W (2010). Xds.. Acta Crystallogr D Biol Crystallogr.

[28] (1994). The CCP4 suite: programs for protein crystallography.. Acta Crystallogr D Biol Crystallogr.

[29] Adams PD, Afonine PV, Bunkoczi G, Chen VB, Davis IW (2010). PHENIX: a comprehensive Python-based system for macromolecular structure solution.. Acta Crystallogr D Biol Crystallogr.

[30] Winn MD, Ballard CC, Cowtan KD, Dodson EJ, Emsley P (2011). Overview of the CCP4 suite and current developments.. Acta Crystallogr D Biol Crystallogr.

[31] Emsley P, Cowtan K (2004). Coot: model-building tools for molecular graphics.. Acta Crystallogr D Biol Crystallogr.

[32] Kleywegt GJ, Brunger AT (1996). Checking your imagination: applications of the free R value.. Structure.

[33] Chen VB, Arendall WB, Headd JJ, Keedy DA, Immormino RM (2010). MolProbity: all-atom structure validation for macromolecular crystallography.. Acta Crystallogr D Biol Crystallogr.

[34] McCoy AJ, Grosse-Kunstleve RW, Adams PD, Winn MD, Storoni LC (2007). Phaser crystallographic software.. J Appl Crystallogr.

[35] Morris GM, Huey R, Lindstrom W, Sanner MF, Belew RK (2009). AutoDock4 and AutoDockTools4: Automated docking with selective receptor flexibility.. J Comput Chem.

